# Word Order and Voice Influence the Timing of Verb Planning in German Sentence Production

**DOI:** 10.3389/fpsyg.2017.01648

**Published:** 2017-09-26

**Authors:** Sebastian Sauppe

**Affiliations:** ^1^Language and Cognition Department, Max Planck Institute for Psycholinguistics, Nijmegen, Netherlands; ^2^Department of Comparative Linguistics, University of Zurich, Zurich, Switzerland

**Keywords:** incremental sentence production, word order, verb planning, German, eye tracking, passive

## Abstract

Theories of incremental sentence production make different assumptions about when speakers encode information about described events and when verbs are selected, accordingly. An eye tracking experiment on German testing the predictions from linear and hierarchical incrementality about the timing of event encoding and verb planning is reported. In the experiment, participants described depictions of two-participant events with sentences that differed in voice and word order. Verb-medial active sentences and actives and passives with sentence-final verbs were compared. Linear incrementality predicts that sentences with verbs placed early differ from verb-final sentences because verbs are assumed to only be planned shortly before they are articulated. By contrast, hierarchical incrementality assumes that speakers start planning with relational encoding of the event. A weak version of hierarchical incrementality assumes that only the action is encoded at the outset of formulation and selection of lexical verbs only occurs shortly before they are articulated, leading to the prediction of different fixation patterns for verb-medial and verb-final sentences. A strong version of hierarchical incrementality predicts no differences between verb-medial and verb-final sentences because it assumes that verbs are always lexically selected early in the formulation process. Based on growth curve analyses of fixations to agent and patient characters in the described pictures, and the influence of character humanness and the lack of an influence of the visual salience of characters on speakers' choice of active or passive voice, the current results suggest that while verb planning does not necessarily occur early during formulation, speakers of German always create an event representation early.

## 1. Introduction

When speakers plan and formulate a sentence, they have to generate a message and transform it into a linearly ordered series of words. This process is generally believed to be incremental, such that speakers can start to articulate an utterance before it is planned in its entirety (Bock and Levelt, 1994; Ferreira and Slevc, 2007, inter alia). There are multiple views of incremental sentence planning that differ in their assumptions about when speakers engage in conceptual encoding and when grammatical structures are built. These views thus also differ in their expectations of when speakers encode information about the event that they are about to describe (i.e., the relations between agents and patients) and when they plan a sentence's verb, expressing the action carried out.

Theoretical accounts of sentence production can be grouped into linearly (or lexically) incremental approaches and hierarchically (or structurally) incremental approaches.

Accounts of linear incrementality assume that sentences are planned “word-by-word” (or phrase-by-phrase) and that speakers begin with the encoding of a nominal message element. The accessibility of message elements is assumed to influence which one is selected first by speakers who begin their sentences by encoding and articulating the most accessible message element's name first. Accessibility is influenced by many factors, two of the most notable are animacy (Branigan et al., 2008) and visual salience (Myachykov et al., 2005; Gleitman et al., 2007). The encoding of other message elements and the event relations between them may be deferred until after speech onset (Gleitman et al., 2007; cf. also Kempen and Hoenkamp, 1987). Linear incrementality thus assumes that, during the piecemeal formulation of utterances, verbs are planned only shortly before they are uttered.

For example, in a series of picture-word interference experiments, Schriefers et al. (1998) found semantic interference effects in German for verbs that were produced early in transitive sentences (in verb-second position) but no interference for sentence-final verbs. This suggests that participants in this study planned verbs before speech onset only when they were positioned near the sentence beginning. Similarly, Allum and Wheeldon (2007) propose that only the first phrase of an utterance is planned before speaking starts. In subject-initial languages this means that the verb may only be planned after articulation of the subject already started.

In contrast, hierarchical incrementality assumes that speakers always generate a representation of the utterance at the outset of formulation. This representation provides “a linguistic action plan that provides information about where to start and how to continue an utterance” (Kuchinsky et al., 2011, p. 749).^1^ The weak version of hierarchical incrementality assumes that planning starts with the encoding of the relationship between agent and patient, i.e., that speakers encode the thematic structure of the event that they are about to describe and what kind of action is carried out. This relational event representation allows speakers then to select a starting point, i.e., to decide which character to mention first (cf. Bock et al., 2004). This view of sentence production was prominently proposed by Griffin and Bock (2000). They report results from a picture description experiment in which English speakers distributed their fixations between agents and patients in an early time window of up to 400 ms after the stimulus pictures appeared on the screen. This initial, pre-linguistic event apprehension phase is assumed to subserve the extraction of “a coarse understanding of the event as a whole” (Griffin and Bock, 2000, p. 274), leading to the construction of a conceptual representation of the utterance, which then guides the linguistic encoding of the sentence's individual words. The results from an eye tracking experiment by Bock et al. (2003) where participants were presented with different clock displays and their task was to tell the time in different formats in Dutch and English also support the hierarchical incrementality. Within a few hundred milliseconds after stimulus onset, speakers had parsed the clocks shown and directed their gaze toward parts in the displays that were relevant for planning the first part of their utterance, supporting the view that linguistic encoding is guided by an utterance representation generated at the outset of formulation (cf. also Kuchinsky et al., 2011). Thus, weakly hierarchically incremental accounts assume that speakers always engage in planning to express the event early by encoding relational information to determine either a rather specific action that is carried out (e.g., *kicking* or *shooting*; Griffin and Bock, 2000) or at least the kind of action or event class (e.g., physical contact event; Bunger et al., 2013).

The strong version of hierarchical incrementality additionally assumes that speakers must engage in some verb planning before articulation of the first word of a sentence can be initiated. Verb planning always entails that the event structure was encoded earlier because speakers need to know about the action and the relations among the participants of a to-be-described event. For example, in Bock and Levelt (1994)'s model of sentence production, verbs play a central role in the planning process by controlling the assignment of syntactic functions, which is served by information contained in the verb lemma about the arguments that each verb takes.

Early evidence for advance verb planning comes from Lindsley (1975) who showed that English speakers needed more time to initiate subject-verb (SV) sentences than to name just the subject when describing pictures; reaction times were also longer for verb-only utterances compared to SV sentences in which the participants already knew the subject. Lindsley argues that these results suggest that verbs are at least partly planned before speech onset. In another picture description study, Kempen and Huijbers (1983) extended Lindsley's account by showing that speakers engage in lexical planning of verbs before speech onset when producing SV sentences.

Ferreira (2000) presents a sentence production model based on Tree-Adjoining Grammar. This model explicitly assumes that lexical selection of verbs is necessary before speakers can plan the first nominal elements of sentences. A verb must be selected before speakers can assign the subject function to one nominal lemma and articulate it sentence-initially because syntactic functions and case marking can only be assigned by verbs. They are assumed to be the syntactic heads of sentences, introducing the necessary structure that is needed to build a syntactic tree which allows the grammatical encoding of subject and object arguments. In support of this account, Ferreira (1994) found that the choice between active and passive voice, i.e., whether the agent or the patient of an event is encoded as the subject, depends on properties of the sentence's verb.

Thus, strongly hierarchically incremental accounts of sentence production assume that speakers always engage in relational encoding and select verbs early during formulation because syntactic function assignment depends on verb lemmas.

Two recent studies have dealt with the advance planning of verbs in verb-final sentences. The first study is Momma et al. (2016), showing that in Japanese verbs are planned before objects. In a picture-word interference experiment, Japanese speakers described pictures with SV or OV sentences while seeing distractor words (superimposed on the pictures) that were either semantically related or unrelated to the verb. Longer speech onset latencies were found for related distractors when speakers produced OV sentences as compared to SV sentences. Momma et al. interpret this as evidence that verb selection occurs before objects but not before subjects because verbs and objects may be more closely associated syntactically than verbs and subjects (cf. Kratzer, 1996).

The second study, Hwang and Kaiser (2014), investigated whether verbs are planned earlier in English (SVO word order) than in Korean (SOV word order) in a combined picture-word interference and eye tracking experiment. Speakers of English and speakers of Korean described pictures of transitive events while hearing auditory distractors that were either unrelated, semantically related to the verb or semantically related to the object (patient). Hwang and Kaiser report longer speech onsets when the distractor was related to the verb in English but not in Korean. There were also differences in the fixation patterns for the two languages: In English, where the verb immediately followed the subject, speakers fixated early (400–600 ms after stimulus onset) on the “action” region, i.e., the part of the picture where agent and patient interacted or were in physical contact and which therefore “provide[s] crucial information about what action is being depicted” (Hwang and Kaiser, 2014, p. 1365). The authors interpret this to be consistent with strongly hierarchically incremental production (called the *lexicalist hypothesis* by Hwang and Kaiser), where verbs are selected already when an utterance plan is generated at the outset of formulation. In Korean, on the other hand, speakers' fixations to the “action” region only increased toward the end of sentences, when the verb was mentioned. This is consistent with weakly hierarchical incrementality which assumes that relational encoding always occurs early but that verbs are planned only shortly before they are articulated; it is, however, also consistent with linearly incremental word-by-word conceptual and linguistic encoding of the sentences. In essence, Hwang and Kaiser interpret their findings as evidence that a language's word order influences when speakers plan the lexical verb during sentence formulation.

The experiment reported here aims to test the centrality of verb planning in sentence production in German by looking at whether voice and word order variations within in a single language may influence when speakers engage in relational encoding and verb planning.

Taking a somewhat different approach than most previous studies, the timing of relational encoding and verb planning during the formulation of German sentences with lexical main verbs placed in different positions is investigated in a simple picture description paradigm. Participants' eye movements were recorded while they spontaneously described pictures of transitive events and the temporal development of fixation preferences before and after speech onset is analyzed.^2^

In German independent declarative sentences, the inflected verb is placed in the second position, i.e., sentence-medially (V-medial). In present or past tense the lexical verb thus immediately follows the first constituent, which is the subject in the current context. However, when the lexical verb occurs as an infinitive or a participle, it is placed sentence-finally (V-final) because the second position is then occupied by another inflected verbal element, e.g., an auxiliary in perfect sentences (*haben* “to have”), a matrix verb (e.g., *versuchen* “to try”) or modal verb (e.g., *sollen* “shall”). Similarly, the lexical verb is also in final position in passive sentences because the second position is occupied by an auxiliary. Also, agents are not arguments but obliques introduced by prepositions in these sentences. These different sentence types are illustrated in Table 1.

**Table 1:** German sentence types relevant for the current experiment.

	*Der*	*Junge*	*tritt*			*den*	*Ball*.
Active, V-medial	Tde	boy	kicks			tde	ball
			“The boy kicks the ball.”				
	*Der*	*Junge*	*hat*		*den*	*Ball*	*getreten*.
	The	boy	has		the	ball	kicked
Active, V-final			“The boy has kicked the ball.”				
	*Der*	*Junge*	*versucht*	*den*	*Ball*	*zu*	*treten*.
	The	boy	tries	the	ball	to	kick
			“The boy tries to kick the ball.”				
	*Der*	*Ball*	*wird*		*vom*	*Jungen*	*getreten*.
Passive	The	ball	is being		by the	boy	kicked
			“The ball is being kicked by the boy.”				

In the current experiment, German speakers described pictures showing transitive, two-participant events using active and passive sentences with V-medial and V-final word orders. Unlike in Hwang and Kaiser (2014), “action” regions are not used here. The reason for this is that it is generally difficult to define regions in the stimulus pictures that exclusively “belong” to the action. Instead, following Norcliffe et al. (2015), relational encoding and verb selection is assumed to manifest itself in the patterns of agent and patient fixations. In an eye-tracked picture description experiment on the Mayan language Tzeltal, Norcliffe et al. showed that speakers distributed their fixations extensively between agents and patients before speech onset when planning verb-initial (verb-patient-agent) sentences. On the other hand, when Tzeltal speakers planned subject-initial (agent-verb-patient) sentences, they preferentially fixated the agent in a time window from the onset of the stimulus picture until speech onset. Norcliffe et al. took these differences to be indicative of early verb planning in verb-initial sentences in Tzeltal. It is thus assumed that the early planning of verbs goes in hand with extensively distributed fixations to agents and patients. Planning verbs requires the encoding of the event relations between referents and it is thus assumed that information about the “action” is mainly distributed between them. A similar approach is also taken by Griffin and Bock (2000) and Konopka and Meyer (2014) who interpret distributed gazes between agents and patients as being indicative of event encoding. Fixation preferences for only one referent before speech onset, or more evenly distributed gazes over both referents, are thus taken to signal different degrees of relational encoding effort and (in the latter case) as being indicative of verb selection.^3^

Given these points, the accounts of hierarchical and linear incrementality make different predictions about when speakers engage in relational event encoding and verb planning in the three different German sentence types in Table 1.

Linearly incremental production accounts assume that speakers do not necessarily start sentence planning by encoding the relations in the described event but may instead start to immediately encode the most accessible nominal concept, which is to be mentioned first (Gleitman et al., 2007). Thus, linear incrementality predicts that the conceptual acceptability or the visual salience of depicted characters influences speakers' choice between producing an active or a passive sentence. Animate or human characters are conceptually more accessible than inanimate or non-human characters and are predicted to be more likely to be chosen as subjects and produced first under this account (Branigan et al., 2008). Visually salient characters are also predicted to be more likely to be selected as subjects because speakers will fixate on them first due to their prominence in the pictures, leading to the encoding of the corresponding character names first (Gleitman et al., 2007).

To ensure fluency in sentences with V-medial word order in which the lexical verb is mentioned immediately after the subject, speakers should engage in extensive relational encoding before speech onset under a linearly incremental account as there might otherwise not be enough time to select and retrieve the verb while uttering the subject (Griffin, 2003). For the production of V-final sentences, speakers may postpone relational and verb encoding until after speech onset because the lexical verb does not have to be ready for articulation so early. It is thus further predicted by linear incrementality that speakers distribute their attention more between agent and patient before speech onset when producing V-medial active sentences than when producing V-final actives and passives, where verb processing can be delayed. Compared to V-medial sentences, more fixations on the subject (the agent in actives and the patient in passives) are expected for V-final sentences because speakers should prioritize lexical encoding of the first referent as they may postpone relational encoding until later.

By contrast, both the weak and the strong version of hierarchical incrementality assume that speakers always encode the event and the relations between referents early in the formulation process in order to generate a conceptual representation of the utterance that guides linguistic encoding. Both versions of hierarchical incrementality predict that the choice between active and passive sentences, and therefore which concept is lexicalized first, may also be influenced by the structure of the event and not only by the conceptual accessibility or visual salience of depicted characters. With respect to verb planning, weakly hierarchical incrementality assumes that speakers encode the action in the depicted event but do not necessarily select a verb early (Griffin and Bock, 2000; Konopka and Meyer, 2014). From this assumption the prediction follows that V-medial and V-final sentences should be associated with different fixation patterns. In order to ensure fluency in the former sentence type (Griffin, 2003), speakers would need to select a verb lemma and begin to linguistically encode the verb before speech onset because it is mentioned immediately after the subject. When formulating V-final sentences, however, speakers may postpone lexical verb selection until after speech onset. Thus, weakly hierarchical incrementality predicts that speakers distribute their visual attention more between agent and patient referents before speech onset during the planning of V-medial as compared to V-final sentences, indicating earlier verb selection in the former. The prediction of differing fixation patterns during the formulation of sentences with different verb positions in weakly hierarchically incremental planning is similar to what is predicted by linear incrementality. However, hierarchical incrementality makes a distinct prediction about the influence of accessibility on grammatical structure choices because this account assumes that the action in the event is encoded early during formulation. Thus, event relations among referents may also be taken into account when speakers choose between producing active or passive sentences.

The strong version of hierarchical incrementality states that relational encoding of the event also always includes or is immediately followed by the selection of a verb lemma, which guides syntactic function assignment (Ferreira, 2000). Therefore, this account predicts that the planning of V-medial and V-final actives as well as passive sentences should all be associated with similar eye movement patterns in which speakers distribute their fixations between agent and patient characters in the pictures, which is assumed to reflect the lexical selection of a verb.

The current experiment tests the predictions of the three different accounts of sentence production by analyzing the influence of agent and patient characters' conceptual accessibility and visual saliency on speakers' choice of active or passive voice when describing pictures of transitive events and by analyzing the patterns of fixations to the characters before and after speech onset using growth curve modeling (Mirman, 2014).

## 2. Methods

### 2.1. Participants

Thirty-three native speakers of German (mean age = 25 years, 10 male) recruited among the (PhD) students of Radboud University and HAN University of Applied Sciences in Nijmegen participated in the experiment. All participants were unaware of the hypotheses of the experiment.

The reported experiment conforms to the American Psychological Association's ethical principle of psychologists and code of conduct (as declared by the ombudsman of the Max Planck Institute for Psycholinguistics). At the time of data collection, ethical approval was not legally required for this kind of study. Written informed consent was obtained from participants at the beginning of the experiment session.

### 2.2. Materials

**Figure 1:**
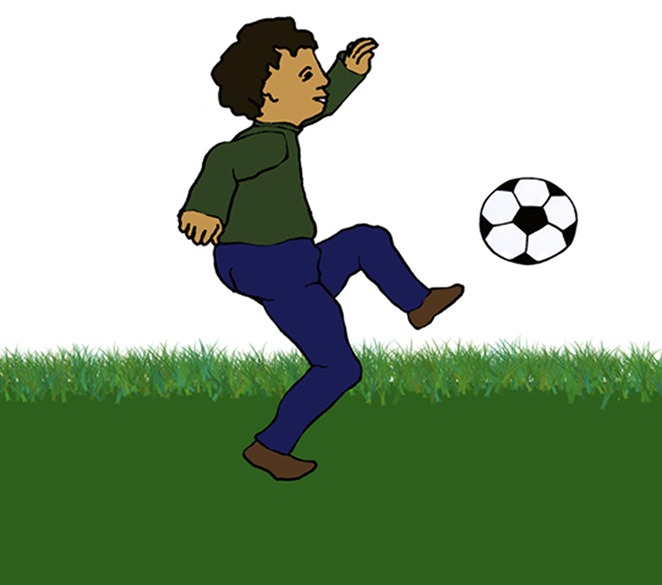
Example stimulus picture.

Colored line drawings of transitive and intransitive events were used as stimuli, including events with both human and non-human agents and patients (cf. Figure 1 for an example). The stimuli are overlapping with the stimuli set of Norcliffe et al. (2015). Target pictures were drawings of 58 transitive events^4^, which were interspersed among 93 filler pictures of intransitive events. Two versions of each target were created by mirror-reversing the pictures. Stimuli were arranged in four lists created by randomizing the order and counterbalancing the two mirror-reversed versions of the targets; at least one filler intervened between any two targets.

### 2.3. Apparatus and procedure

Stimuli were presented on a Tobii T120 eye tracker (resolution = 1,024 × 768 pixels, sampling rate = 60 Hz, distance to participant ≈ 58–60 cm). Eye data and vocal responses were recorded together by the Tobii Studio software. Fixations and saccades were defined by the Tobii I-VT filter (Olsen, 2012). Areas of interest (AOIs) were defined covering the agent and patient characters and a slim margin around them in the stimulus pictures (Holmqvist et al., 2011). Testing took place in a dimly lit and soundproof booth.

The participants' task was to describe the pictures in one sentence as quickly and accurately as possible, naming all depicted characters. Each stimulus was preceded by a fixation dot appearing randomly in one out of five positions at the top of the screen. When participants fixated on the dot, the experimenter (who monitored their gaze on the computer controlling the eye tracker) initiated the next trial, making sure that their gaze did not fall on the agent or patient when the stimulus appeared. The experiment started with a practice phase in which 15 example pictures with accompanying pre-recorded descriptions were presented to participants in order to familiarize them with the task. Next, the same pictures were presented again one at a time and participants were asked to describe them themselves. The testing phase followed in which stimuli were presented in three blocks lasting 8–10 min each. Calibration was performed at the beginning of each block.

### 2.4. Sentence scoring and data selection

Utterances produced on target trials were transcribed and scored as active or passive sentences or as other constructions (e.g., existentials). For each target trial, the onset and offset of each word were annotated manually in Praat (Boersma, 2001). Only actives and passives with both referents realized overtly were included in the analyses. Trials in which participants corrected themselves were excluded. However, when the response contained disfluencies (like “uh”) or pauses it was still included because these are normal phenomena occurring during spontaneous speaking. Trials were also excluded when the first fixation fell on the agent or patient instead of the fixation dot, if the first fixation to agent or patient occurred later than 600 ms after picture onset, and where two consecutive fixations were longer than 600 ms apart (indicating track loss). Additionally, trials where excluded if speech onset was longer than 6,500 ms or longer than three standard deviations away from the grand mean. Two stimulus pictures did not elicit any responses conforming to these criteria and were thus excluded from the analyses. Two trials with semantic role reversals, i.e., where participants conceptualized the event so that the intended patient served as the agent and the intended agent as the patient, were included and recoded accordingly. The final data set consisted of 1,207 sentences [active, V-medial: 922 sentences, 6 with disfluencies; active, V-final: 180 sentences (including 23 sentences with matrix verbs, cf. Table 1); passive: 105 sentences, 1 with a disfluency]. Figure S1 in the Supplementary Materials shows how the different sentence types were distributed among stimuli.

### 2.5. Analyses

A generalized linear mixed effects regression model was employed to analyze which variables influenced voice choice. This model included as predictors the humanness of agent and patient (conceptual accessibility) and which referent was looked at first (visual saliency). Humanness was chosen over animacy as predictor because only few sentences with inanimate agents were included in the dataset.

To examine the time course of relational encoding and verb selection, the likelihoods of fixations to first and second referents (subjects and objects/obliques) were analyzed with logistic growth curve regression (Mirman, 2014; Donnelly and Verkuilen, 2017). Growth curve analysis is a variety of linear mixed effects regression that uses orthogonalized polynomial time terms as predictors to describe the major aspects of the observed fixation curve shapes.

The overall development of gaze patterns was assessed in two analysis time windows. Within each time window, fixations were aggregated into 200 ms bins for each trial to reduce statistical non-independencies in eye tracking data (temporal autocorrelation; Barr, 2008). These non-independencies result from the fact that eye gaze cannot change location instantaneously but must rather “travel through time and space” (Barr, 2008, p. 464), making participants' eye movement behavior at one time step highly correlated with that at the next time step. Aggregation of fixations into trial-wise time bins helps to filter “out the eye-movement based dependencies” (Barr, 2008, p. 464). Additionally, for each time bin in each trial, the number of samples with fixations to the agent or the patient in the previous time bin was included as a nuisance variable (cf. Sassenhagen and Alday, 2016) with the aim of further reducing temporal autocorrelation which is due to the fact that eye movements are relatively slow as compared to the sampling rate of the eye tracker (Duchowski, 2007; Barr, 2008). Fixation likelihoods were calculated on the basis of all fixations to agent and patient AOIs as well as fixations to “whitespace” (Holmqvist et al., 2011) outside of these AOIs.

The first analysis time window spanned the time between presentation of the stimulus and grand mean speech onset (100–1,700 ms, grand mean speech onset = 1,712 ms). Fixations during the first 100 ms were not included because eye movements in response to stimulus presentation are unlikely to occur this early (Duchowski, 2007). The second time window reached from grand mean speech onset until 200 ms before grand mean speech offset by which most planning should have been finished (1,700–3,500 ms; grand mean speech offset = 3,732 ms).

Sentence Type and fourth-order orthogonalized polynomial time terms were included as predictors in all regressions (Mirman et al., 2008; Mirman, 2014). Each of the included polynomial time terms describes a different aspect of the eye movement data (cf. Kalénine et al., 2012). Linear time (Time^1^) describes the angle or the slope of fixation curves, with more positive predictor estimates indicating a steeper increase over the course of the analysis time window. Quadratic time (Time^2^) describes the rate of increases and decreases in the form of a parabolic curve, where more positive estimates indicating a more “U-shaped” curve and more negative estimates describing curves with “inverted U-shapes”. Cubic time (Time^3^) describes earlier or later increases or decreases of the fixation curves, i.e., how “S-shaped” the fixation curves are. Finally, quartic time (Time^4^) describes secondary peaks in the curves' tails, at the beginning or end of the analysis time window. A main effect of Sentence Type in the growth curve regression models means that one sentence type exhibited overall higher or lower fixation likelihoods to a character in the analysis time window than the other sentence type. Interaction effects between Sentence Type and the polynomial time terms mean that fixation likelihoods changed differently over the course of the analysis time window in different sentence types, i.e., that the fixation curves exhibit different shapes.

Two further nuisance variables and their interactions with the time terms were included in order to control for their effects on speakers' likelihood to fixate on subject and object characters during sentence formulation. First, speech onset latencies were included to account for variations in fixation patterns that are reducible to sentence formulation processes with different timing across trials, independently of the produced sentence type. For example, if the speech onset in one trial is earlier than the grand mean of 1,712 ms, the speaker might look away from the subject character already earlier than in trials with longer speech onsets, leading to potentially different fixation curves.

Second, event codability was also included as a nuisance variable. This variable describes to what degree speakers use the same verbs to describe an event, which is related to the difficulty of recognizing the depicted action (Kuchinsky, 2009). It is characterized as reflecting “consensus […] about the conceptual structure of an event” by (van de Velde et al., 2014, p. 125). In other words, the codability describes how “easy” or “hard” it was for speakers to find an appropriate verb to name the depicted event. By manipulating event codability in a picture description experiment, van de Velde et al. show that speakers distributed their visual attention more between agent and patient if the event was highly codable (i.e., it is “easy” to name the verb) but focused more in the first-mentioned character when event codability was low. The aim of the current experiment is to test differences in eye movement patterns in order to examine whether planning time courses differences between sentence types with different voice marking and word order. Including event codability as nuisance variable in the regression models accounts for potential differences in fixation patterns that might be explained solely by different planning strategies employed for highly and lowly codable events. To assess the event codability of each stimulus picture, the Shannon entropy *H* (Shannon, 1948) was computed, describing the variability in which verbs participants used in their responses. If it was difficult to recognize the event depicted in a picture and participants therefore produced many different verbs, that picture's *H* was larger (low codability) than that of a picture where participants highly agreed on which verb to choose (high codability).

Speech onset latencies were analyzed using a linear mixed effects regression model predicting log-transformed reaction times. In addition to the predictors used in the analysis of voice choice (humanness of agent and patient characters and the identity of the first-fixated character), sentence type was a further predictor in this model. Event codability was also included as a nuisance variable in order to control for effects on speech onset latencies caused the relative ease of finding a verb to describe the event.

Significance of fixed effects was assessed with Wald *Z* tests in generalized linear regression models (Agresti, 2007; Jaeger, 2008) and with Type II Wald *F*-tests with Kenward-Roger approximation of degrees of freedom in the linear regression model for speech onsets (Kenward and Roger, 1997; Halekoh and Højsgaard, 2014). Where these variables were included, sentence type was Helmert-coded and all other categorical variables were contrast-coded (Cohen et al., 2003). The maximal random effects structure justified by design (that allowed convergence) was used for all models (Barr, 2013; Barr et al., 2013). All models were computed using the *lme4* package in R (Bates et al., 2015; R Core Team, 2015). Graphs were produced using *ggplot2* (Wickham, 2009).

## 3. Results

**Figure 2:**
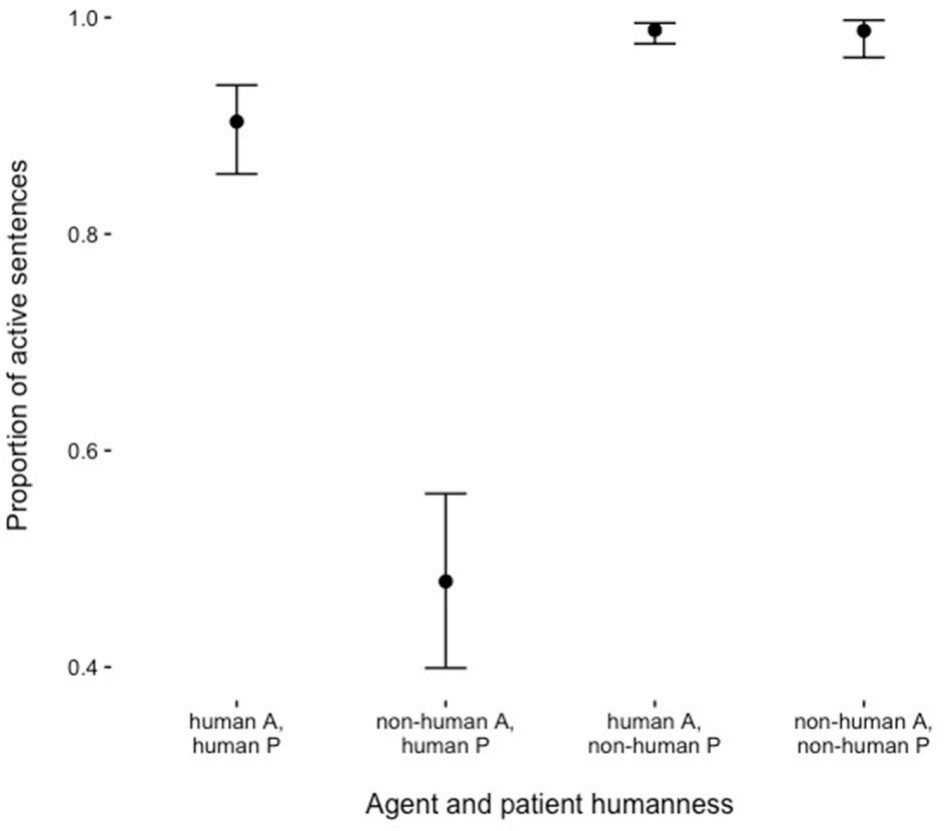
Proportions of active sentences as a function of agent (A) and patient (P) humanness. Bars indicate 95% confidence intervals (Agresti and Coull, 1998).

Figure 2 shows the proportion of active sentences produced as a function of the humanness of agent and patient characters. In general, participants were more likely to produce passives when the patient was human (main effect of Patient Humanness in Table 2). In addition, passives were more likely to be produced in the descriptions of stimulus pictures with non-human agents and human patients. The identity of the first-fixated character in the pictures had no statistically significant influence on voice choice.

**Table 2:** Results from binomial generalized linear mixed effects regression model predicting voice choice.

	$\hat{\beta }$	**|*Z*|**	***p***	
Intercept	−5.69	6.61	< 0.001	^***^
Agent humanness (=non-human)	0.19	0.24	0.81	
Patient humanness (=non-human)	−7.92	5.88	< 0.001	^***^
Agent humanness × patient humanness	−6.04	3.57	< 0.001	^***^
First-fixated character (=patient)	0.73	1.54	0.12	

*** *p < 0.001*.

**Figure 3:**
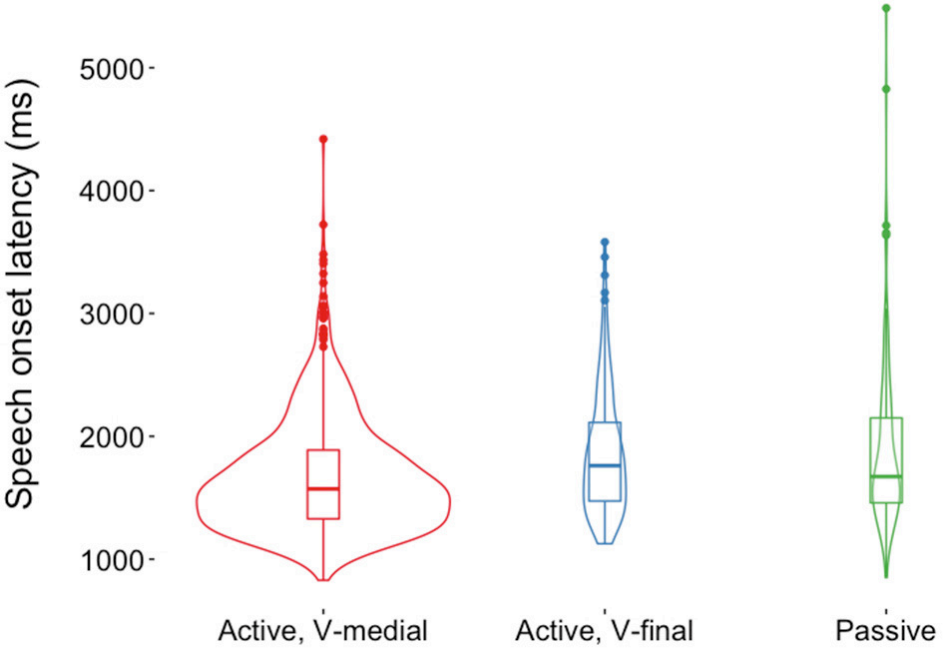
Densities and box plots of speech onset latencies (relative to stimulus picture onset) for three German sentence types; width of the violins is proportional to the number of underlying data points (Hintze and Nelson, 1998).

Figure 3 shows the distribution of speech onset latencies for each sentence type. Speech onset latencies were numerically shorter for V-medial actives (mean = 1,662 ms, *SD* = 458 ms) than for V-final actives (mean = 1,859 ms, *SD* = 504 ms) and passives (mean = 1,912 ms, *SD* = 722 ms). However, there were no statistically significant effects on speech onset latencies (all *p*s > 0.14, Table 3). Sentence types did also not differ with respect to the onset of the verb or auxiliary after the subject or the onset of the second NP (all *p*s > 0.23 in a linear mixed effects regressions with Sentence Type as predictor, cf. Tables S1, S2). This indicates that the occurrence of pauses was not systematically related to voice or word order in the current dataset. Thus, it is justified to compare participants' eye movements during the production of V-medial actives, V-final actives and passive sentences in the same analysis time windows because speech onset times, phrase durations and the distribution of pauses and disfluencies was similar across sentence types.

**Table 3:** Results from linear mixed effects regression model predicting log-transformed speech onset latencies.

	$\hat{\beta }$	**|*t*|**	***F* statistic**	***p***
Intercept	7.45	230.54		
Agent humanness (= non-human)	> −0.01	0.07	*F*_(1, 41)_ = 0.03	0.87
Patient humanness (= non-human)	−0.03	1.00	*F*_(1, 57)_ = 0.69	0.41
Agent humanness × patient humanness	−0.02	0.29	*F*_(1, 30)_ = 0.06	0.81
Actives vs. passives	−0.04	0.97	*F*_(2, 27)_ = 2.08	0.14
V-final actives vs. V-medial actives	0.05	1.93		
First-fixated character (= patient)	−0.02	1.34	*F*_(1, 31)_ = 1.49	0.23
Event codability (*z*-transformed)	0.01	1.03	*F*_(1, 33)_ = 0.69	0.41

**Figure 4:**
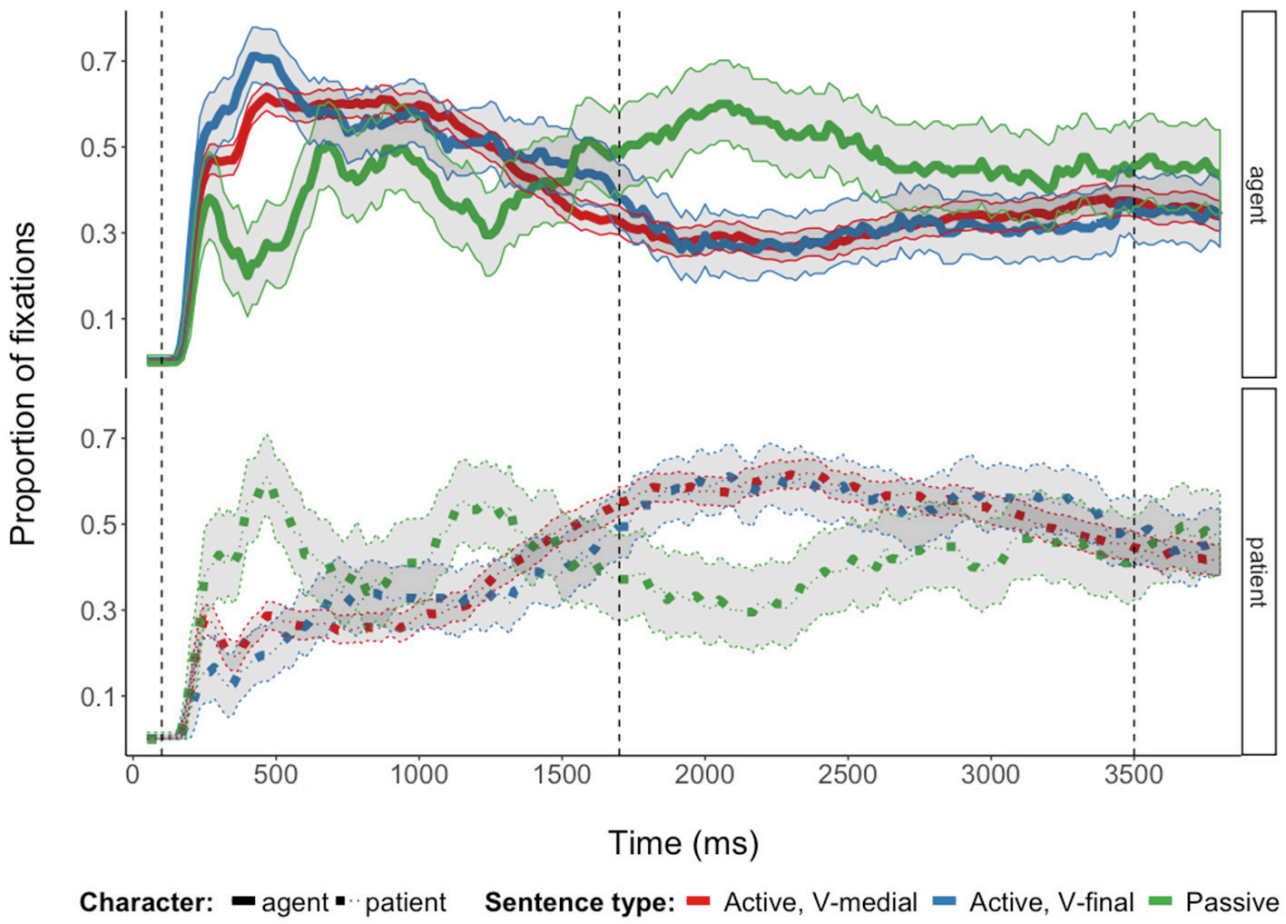
Proportions of fixations to agents and patients during the production of three German sentence types. Proportions are based on fixations to agent and patient AOIs and to “whitespace” (Holmqvist et al., 2011) not covered by these AOIs. Ribbons indicate 95% multinomial confidence intervals (Sison and Glaz, 1995; Villacorta, 2012); vertical lines indicate analysis time windows.

In all sentence types (actives with V-medial and V-final word order and passives), speakers concentrated their gazes on the first mentioned referent until shortly before speech onset when they switched to the second mentioned referent (Figure 4; cf. also Figure S2). Toward the end of the sentences, proportions of fixations to the two referents approximated each other (cf., e.g., Griffin and Bock (2000), Konopka and Meyer (2014) and Norcliffe et al. (2015) for similar fixation patterns). Despite these overall similarities, speakers' gaze behavior differed between sentence types.

When planning and producing passive sentences, speakers distributed their visual attention more evenly between characters then when planning active sentences in general. Before speech onset, fixations to the subject character (agent in actives, patient in passives) showed an earlier and steeper increase and decrease before speech onset in passives (interactions of Sentence Type and Time^2^ and Time^3^ in 100–1,700 ms time window, Table 4). Speakers' fixations to object/oblique characters (patient in actives, agent in passives) also increased earlier in passive sentences than in actives (interaction of Sentence Type and Time^3^ in 100–1,700 ms time window). After speech onset, the more even distribution of visual attention between subject and object/oblique characters is revealed by overall fewer and more quickly declining looks to the agent in passives as compared to the patient in actives (main effect of Sentence Type and interactions between Sentence Type and Time^1^ and Time^2^ in 1,700–3,500 ms time window, regression model for object/oblique fixations in Table 4). Fixations to the subject character increased earlier again after speech onset in passives as compared to actives (interactions between Sentence Type and Time^3^ and Time^4^ in 1,700–3,500 ms time window).

**Table 4:** Results from binomial generalized linear mixed effects regression models predicting subject and object/oblique fixations in V-medial actives, V-final actives and passive sentences.

	**Subject fixations**				**Object/oblique fixations**			
	$\hat{\beta }$	|*Z*|	*p*		$\hat{\beta }$	|*Z*|	*p*	
**100–1,700 ms**								
Intercept	−0.59	4.09	<0.001	^***^	−1.89	10.32	<0.001	^***^
Time^1^	−1.21	2.33	0.02	^*^	2.38	4.39	<0.001	^***^
Time^2^	−2.98	5.09	<0.001	^***^	−1.11	2.54	0.01	^*^
Time^3^	−1.27	2.31	0.02	^*^	3.06	6.29	<0.001	^***^
Time^4^	−1.23	3.92	<0.001	^***^	−0.58	1.54	0.12	
Actives vs. passives	−1.19	3.69	<0.001	^***^	1.06	3.06	<0.01	^**^
V-final actives vs. V-medial actives	−0.21	0.79	0.43		−1.43	4.18	<0.001	^***^
Time^1^ × Actives vs. passives	0.69	0.61	0.55		1.90	1.32	0.18	
Time^1^ × V-final actives vs. V-medial act.	−1.58	1.64	0.10		1.86	2.08	0.04	^*^
Time^2^ × Actives vs. passives	−2.73	2.22	0.03	^*^	1.61	1.18	0.24	
Time^2^ × V-final actives vs. V-medial actives	−2.21	1.97	<0.05	^*^	−1.76	1.72	0.09	
Time^3^ × Actives vs. passives	−3.47	2.47	0.01	^*^	2.75	2.62	<0.01	^**^
Time^3^ × V-final actives vs. V-medial actives	−1.14	1.79	0.07		2.18	2.29	0.02	^*^
Time^4^ × Actives vs. passives	−0.02	0.04	0.96		1.27	1.46	0.14	
Time^4^ × V-final actives vs. V-medial actives	−1.54	2.80	<0.01	^**^	−0.25	0.35	0.72	
Fixations to AOI in previous time bin	0.19	101.74	<0.001	^***^	0.21	97.39	<0.001	^***^
Speech onset latency (*z*-transformed)	0.28	27.49	<0.001	^***^	−0.25	19.46	<0.001	^***^
Time^1^ × Speech onset latency	0.88	26.80	<0.001	^***^	−0.77	20.49	<0.001	^***^
Time^2^ × Speech onset latency	0.31	9.46	<0.001	^***^	−0.41	11.14	<0.001	^***^
Time^3^ × Speech onset latency	−0.49	14.94	<0.001	^***^	0.49	13.49	<0.001	^***^
Time^4^ × Speech onset latency	−0.41	13.05	<0.001	^***^	0.39	11.05	<0.001	^***^
Event codability (*z*-transformed)	−0.06	1.19	0.23		−0.08	1.42	0.16	
Time^1^ × Event codability	−0.28	2.72	<0.01	^**^	0.46	2.67	<0.01	^**^
Time^2^ × Event codability	0.03	0.39	0.70		−0.37	1.90	0.06	
Time^3^ × Event codability	−0.07	0.58	0.56		0.33	2.32	0.02	^*^
Time^4^ × Event codability	0.44	4.60	<0.001	^***^	−0.56	4.72	<0.001	^***^
**1,700–3,500 ms**								
Intercept	−1.52	6.35	<0.001	^***^	0.11	0.44	0.66	
Time^1^	1.61	2.50	0.01	^*^	−1.59	2.80	<0.01	^**^
Time^2^	−1.44	2.20	0.03	^*^	1.57	2.36	0.02	^*^
Time^3^	1.88	3.19	<0.01	^**^	−0.73	1.30	0.19	
Time^4^	1.48	3.34	<0.001	^***^	0.82	2.08	0.04	^*^
Actives vs. passives	0.33	0.51	0.61		−1.35	2.09	0.04	^*^
V-final actives vs. V-medial actives	−0.92	2.97	<0.01	^**^	0.52	1.06	0.29	
Time^1^ × Actives vs. passives	3.50	1.83	0.07		−3.38	2.26	0.02	^*^
Time^1^ × V-final actives vs. V-medial act.	0.07	0.08	0.94		0.36	0.39	0.69	
Time^2^ × Actives vs. passives	−2.53	1.34	0.18		3.52	2.02	0.04	^*^
Time^2^ × V-final actives vs. V-medial actives	−1.09	1.96	0.05		0.99	1.00	0.32	
Time^3^ × Actives vs. passives	5.22	3.11	<0.01	^**^	−0.97	0.68	0.50	
Time^3^ × V-final actives vs. V-medial actives	0.38	0.49	0.63		−0.81	0.76	0.45	
Time^4^ × Actives vs. passives	3.00	2.59	<0.01	^**^	−1.81	1.79	0.07	
Time^4^ × V-final actives vs. V-medial actives	0.57	0.98	0.33		−0.55	1.00	0.32	
Fixations to AOI in previous time bin	0.30	164.74	<0.001	^***^	0.29	173.84	<0.001	^***^
Speech onset latency (*z*-transformed)	−0.04	3.25	<0.01	^**^	0.11	8.88	<0.001	^***^
Time^1^ × Speech onset latency	−0.49	12.84	<0.001	^***^	0.60	16.93	<0.001	^***^
Time^2^ × Speech onset latency	0.32	8.63	<0.001	^***^	−0.26	7.28	<0.001	^***^
Time^3^ × Speech onset latency	−0.05	1.45	0.15		0.07	2.30	0.02	^*^
Time^4^ × Speech onset latency	−0.01	0.28	0.78		<0.01	0.03	0.98	
Event codability (*z*-transformed)	0.15	2.69	<0.01	^**^	−0.10	1.43	0.15	
Time^1^ × Event codability	0.12	0.99	0.32		−0.26	2.58	<0.01	^**^
Time^2^ × Event codability	0.11	1.30	0.19		−0.03	0.34	0.73	
Time^3^ × Event codability	0.16	1.36	0.17		0.15	1.04	0.30	
Time^4^ × Event codability	−0.11	1.21	0.23		0.20	1.91	0.06	

* *p < 0.05*,

** *p < 0.01*,

*** *p < 0.001*.

Crucially, there were also differences in fixation patterns for the planning and formulation of active sentences with different word orders. During the production of V-medial actives, where the verb was mentioned immediately after the subject, speakers looked at the subject (agent) with a steeper increase and decrease before speech onset than in V-final actives (interaction of Sentence Type and Time^2^ in 100–1,700 ms time window, Table 4). In addition, there were slightly more subject fixations at the beginning of the analysis time window in V-medial actives, which is captured by the significant interaction between Sentence Type and Time^4^. Visual inspection of the fitted values from the regression model supports this interpretation of the quadratic and quartic differences between V-medial and V-final actives (Figure S3). After speech onset, there were overall more fixations directed toward the subject character when speakers produced V-final sentences. Actives with V-medial and V-final word orders differed also with respect to fixations to the object (patient) characters before speech onset. Object fixations increased steeper but with a flatter curve shape for V-medial actives than for V-final actives before speech onset (interactions of Sentence Type and Time^1^ and Time^3^ in 100–1,700 ms time window, Table 4). Active sentence types did not differ in object fixations after speech onset.

## 4. Discussion and conclusions

The current experiment yielded three main findings: First, there were differences in gaze patterns for active sentences with V-medial and V-final word order. Second, German speakers fixation behavior before speech onset also differed for the production of active and passive sentences. Third, the choice of passives was largely determined by patient humanness and the relative humanness of agent and patient.

The fixation differences that were found between V-medial and V-final actives suggest that verb planning was influenced by the position of the verb in the sentence. When the verb was in an early position and thus would be articulated immediately after the subject in V-medial actives, German speakers distributed their attention more between agent (subject) and patient (object) before speech onset. Put differently, speakers looked more at the subject and less at the object before speech onset in V-final actives as compared to V-medial actives, suggesting that they concentrated more on lexical encoding of the subject before speech onset in verb-final sentences.^5^ This finding is incompatible with the predictions made by the strong version of hierarchical incrementality (Bock and Levelt, 1994; Ferreira, 2000). If verb selection was a prerequisite to assign syntactic functions and prepare the sentence's subject, speakers should have distributed their visual attention between agents and patients in similar ways in all sentence types, independently of the position of the verb. The differences in fixation patterns between V-medial and V-final actives are compatible with linear incrementality, which predicted that sentence planning proceeds word-by-word and thus that verbs are only planned shortly before they will be articulated (Gleitman et al., 2007). They are also compatible with the weak version of hierarchical incrementality, which predicted that first a structural-relational utterance representation is generated that guides linguistic encoding, which is carried out in the order in which words will be uttered. This would lead sentence-medial verbs to be prepared earlier than sentence-final verbs (Griffin and Bock, 2000; Kuchinsky et al., 2011).

When formulating passives, speakers distributed their visual attention even more between agent and patient before speech onset than when formulating active sentences. Figure 4 shows that speakers first primarily fixated on the patient referent (the subject of these sentences) before fixating on the agent (realized as oblique); toward the end of the 100–1,700 ms time window the patient was again fixated primarily by the participants. This suggests that planning passives required more relational encoding than active planning and the reason for this could be that passives often describe non-prototypical animacy configurations in which a human is acted upon, that they are less frequent, and that the planning operations involved potentially differ from those of actives (Sauppe, 2017). Potentially different planning operations between actives and passives might also account for why speakers still distributed their visual attention more evenly between characters in passives also after speech onset, for example due to the affordances of preparing passivized verb forms.

Additionally, the humanness of patient characters as well as the patient and the agent characters' relative humanness influenced the choice between the production of active and passive voice marking. This shows that speakers did not simply assign the subject function to conceptually highly accessible human referents but analyzed the depicted event and the semantic roles and humanness of referents early in the formulation process in order to guide their structural choices (cf. Dobel et al., 2007; Hafri et al., 2013). In a picture description experiment on German, van Nice and Dietrich (2003) also found that agent and patient animacy influenced the choice between active and passive sentences. The current effect of humanness, which is contingent on semantic roles, supports hierarchically incremental accounts of sentence production (Kuchinsky et al., 2011), which propose that planning starts with the generation of an utterance plan that includes the relations among event participants. The effect is thus incompatible with linearly incremental accounts.

Altogether, the current results support weakly hierarchically incremental accounts of sentence production (Griffin and Bock, 2000; Konopka and Meyer, 2014). The differences in fixation patterns between V-medial and V-final active sentences suggest that verbs are only selected early when they were mentioned immediately after the subject and the finding that the choice of active vs. passive was driven by the relative humanness of the patient and the agent (and not just by humanness in general) indicates that speakers always encoded the event early to assess the semantic roles of the depicted characters. Thus, while speaking may start without the selection of a verb lemma (Iwasaki, 2011), the formulation of sentences still appears to depend on a representation of the described event instead of being solely driven by the retrieval of individual words (Norcliffe et al., 2015). These representations may be sufficient to assign syntactic functions, without the need to completely encode the verb first (in the case of verb-final active sentences). In general, differences in fixation patterns between active and passive sentences arose shortly after the stimulus pictures were presented, indicating that participants decided on the structure of the to-be-produced sentence early in the formulation process (Griffin and Bock, 2000).

The differences in the timing of verb planning between V-medial and V-final active sentences in German are similar to the differences that Hwang and Kaiser (2014) found between English and Korean. A weakly hierarchically incremental production account can explain the finding that speakers generated a representation of the event early but at the same time only engaged in additional extensive relational encoding and verb planning early when the verb was positioned sentence-medially.

It is an open question whether the event representations that German speakers appeared to have generated at the outset of sentence formulation to choose between active and passive sentence structures are “impoverished” or whether they are homologous to the utterance plans that are assumed to be generated at the beginning of the planning process in accounts of hierarchical incrementality (Griffin and Bock, 2000). The event representations must minimally contain information about the semantic roles of event participants and about their humanness. Utterance plans, however, are also assumed to contain more detailed information about the type of the event and a structural representation of the sentence under production (Bock and Ferreira, 2014).

In sum, the current experiment provides a temporally fine-grained view of verb planning in unscripted German sentence production, suggesting that the generation of an event representation is a necessary pre-requisite to start speaking, but not the retrieval of a verb, especially when it is positioned sentence-finally. The results reported here are consistent with the findings of Schriefers et al. (1998) on German and Momma et al. (2016) on Japanese, who demonstrated that speakers do not have to select sentence-final verbs before they can initiate the articulation of subjects.

The scope and time course of sentence planning may be influenced by many factors, ranging from time pressure (Ferreira and Swets, 2002) and speakers' working memory capacity (Swets et al., 2014) to prior knowledge about the event and ease of event encoding (van de Velde et al., 2014; Konopka and Kuchinsky, 2015). Here, it was shown that just as differences in grammars may lead to different planning preferences across languages (Norcliffe and Konopka, 2015), word order and voice variations can also influence the timing of relational encoding and verb planning within a language.

## Author contributions

The author confirms being the sole contributor of this work and approved it for publication.

### Conflict of interest statement

The author declares that the research was conducted in the absence of any commercial or financial relationships that could be construed as a potential conflict of interest.

## Footnotes

^1^The idea of hierarchically incremental sentence planning goes back to Wilhelm Wundt's *Die Sprache* (Wundt, 1900), where he proposed that speakers begin sentence formulation with a *Gesamtvorstellung* (“total image”) of an event, which is then dissected into its parts—entities, properties, and states—, allowing them to be lexicalized and uttered (Levelt, 2013).

^2^Pupillometric data from this experiment are reported in Sauppe (2017).

^3^Note that hypotheses on speakers' eye movement behaviors refer to the patterns that emerge when looking at the mean across trials.

^4^Only pictures of transitive events were included as target items to ensure that all critical trials exhibited similar “relational complexities” in the sense of involving two participants, agent and patient. Momma et al. (2016, p. 820) acknowledge that the difference in transitivity between their SV (intransitive) and OV (transitive) conditions might have also contributed to planning differences.

^5^Based on the results of the growth curve analysis (interaction between sentence type and the quadratic and quartic time terms) and the proportions of fixations (Figure 4) it may be speculated that the (arguably small) differences between V-medial and V-final actives are manifested at the beginning and the end of the 100–1,700 ms analysis time window, defined by the presentation of the stimulus picture and grand mean speech onset (cf. also Figures S2, S3). One possible explanation for this is that the more evenly distributed fixations among agents and patients for V-medial actives shortly after stimulus presentation reflect that speakers primarily engaged in relational encoding and verb selection to prepare the verb early in the production of these sentences, deciding very quickly on what kind of sentence they would produce. After these initial differences (presumably resulting from earlier verb planning), speakers might have concentrated on the encoding of the subject character in both active sentence types. The fixation proportions in Figure 4 suggest that this was followed again by slightly more distributed agent (subject) and patient (object) fixations in V-medial sentences; this can be interpreted as a consequence of speakers' need to begin preparing the word forms of verbs already before speech onset when they were mentioned immediately after the subject to avoid disfluencies (cf. Griffin, 2001, 2003), whereas in V-final sentences this was not necessary before speech onset.

## References

[B1] Agresti A. (2007). An Introduction to Categorical Data Analysis, 2nd Edn. Hoboken, NJ: John Wiley and Sons 10.1002/0470114754

[B2] Agresti A. Coull B. A. (1998). Approximate is better than “exact” for interval estimation of binomial proportions. Amer. Stat. 52, 119–126.

[B3] Allum P. H. Wheeldon L. R. (2007). Planning scope in spoken sentence production: the role of grammatical units. J. Exp. Psychol. 33, 791–810. 10.1037/0278-7393.33.4.791 17576154

[B4] Barr D. J. (2008). Analyzing ‘visual world’ eyetracking data using multilevel logistic regression. J. Mem. Lang. 59, 457–474. 10.1016/j.jml.2007.09.002

[B5] Barr D. J. (2013). Random effects structure for testing interactions in linear mixed-effects models. Front. Psychol. 4:328. 10.3389/fpsyg.2013.00328 23761778 PMC3672519

[B6] Barr D. J. Levy R. Scheepers C. Tily H. J. (2013). Random effects structure for confirmatory hypothesis testing: keep it maximal. J. Mem. Lang. 68, 255–278. 10.1016/j.jml.2012.11.001 24403724 PMC3881361

[B7] Bates D. Mächler M. Bolker B. Walker S. (2015). Fitting linear mixed-effects models using lme4. J. Stat. Softw. 67, 1–48. 10.18637/jss.v067.i01

[B8] Bock K. Ferreira V. S. (2014). Syntactically speaking, in The Oxford Handbook of Language Production, eds Goldrick M. Ferreira V. S. Miozzo M. (Oxford: Oxford University Press), 21–46.

[B9] Bock K. Irwin D. E. Davidson D. J. (2004). Putting first things first, in The Interface of Language, Vision and Action: Eye Movements and the Visual World, eds Henderson J. M. Ferreira F. (New York; Hove: Psychology Press), 249–278.

[B10] Bock K. Irwin D. E. Davidson D. J. Levelt W. J. M. (2003). Minding the clock. J. Mem. Lang. 48, 653–685. 10.1016/S0749-596X(03)00007-X

[B11] Bock K. Levelt W. J. M. (1994). Language production: grammatical encoding, in Handbook of Psycholinguistics, 1st Edn., ed Gernsbacher M. A. (San Diego, CA: Academic Press), 945–984.

[B12] Boersma P. (2001). Praat, a system for doing phonetics by computer. Glot Int. 5, 341–345.

[B13] Branigan H. P. Pickering M. J. Tanaka M. N. (2008). Contributions of animacy to grammatical function assignment and word order during production. Lingua 118, 172–189. 10.1016/j.lingua.2007.02.003

[B14] Bunger A. Papafragou A. Trueswell J. C. (2013). Event structure influences language production: evidence from structural priming in motion event description. J. Mem. Lang. 69, 299–323. 10.1016/j.jml.2013.04.002 24072953 PMC3780438

[B15] Cohen J. Cohen P. West S. G. Aiken L. S. (2003). Applied Multiple Regression/Correlation Analysis for the Behavioral Sciences, 3rd Edn. Mahwah, NJ: Lawrence Erlbaum Associates.

[B16] Dobel C. Gumnior H. Bölte J. Zwitserlood P. (2007). Describing scenes hardly seen. Acta Psychol. 125, 129–143. 10.1016/j.actpsy.2006.07.004 16934737

[B17] Donnelly S. Verkuilen J. (2017). Empirical logit analysis is not logistic regression. J. Mem. Lang. 94, 28–42. 10.1016/j.jml.2016.10.005

[B18] Duchowski A. (2007). Eye Tracking Methodology: Theory and Practice, 2nd Edn. London: Springer.

[B19] Ferreira F. (1994). Choice of passive voice is affected by verb type and animacy. J. Mem. Lang. 33, 715–736. 10.1006/jmla.1994.1034

[B20] Ferreira F. (2000). Syntax in language production: an approach using tree-adjoining grammars, in Aspects of Language Production, ed Wheeldon L. R. (Philadelphia, PA: Psychology Press), 291–330.

[B21] Ferreira F. Swets B. (2002). How incremental is language production? Evidence from the production of utterances requiring the computation of arithmetic sums. J. Mem. Lang. 46, 57–84. 10.1006/jmla.2001.2797

[B22] Ferreira V. S. Slevc L. R. (2007). Grammatical encoding, in The Oxford Handbook of Psycholinguistics, ed Gaskell M. G. (Oxford: Oxford University Press), 453–469.

[B23] Gleitman L. R. January D. Nappa R. Trueswell J. C. (2007). On the *give* and *take* between event apprehension and utterance formulation. J. Mem. Lang. 57, 544–596. 10.1016/j.jml.2007.01.007 18978929 PMC2151743

[B24] Griffin Z. M. (2001). Gaze durations during speech reflect word selection and phonological encoding. Cognition 82, B1–B14. 10.1016/S0010-0277(01)00138-X 11672707 PMC5130081

[B25] Griffin Z. M. (2003). A reversed word length effect in coordinating the preparation and articulation of words in speaking. Psychon. Bull. Rev. 10, 603–609. 10.3758/BF03196521 14620353 PMC5533605

[B26] Griffin Z. M. Bock K. (2000). What the eyes say about speaking. Psychol. Sci. 11, 274–279. 10.1111/1467-9280.00255 11273384 PMC5536117

[B27] Hafri A. Papafragou A. Trueswell J. C. (2013). Getting the gist of events: recognition of two-participant actions from brief displays. J. Exp. Psychol. 142, 880–905. 10.1037/a0030045 22984951 PMC3657301

[B28] Halekoh U. Højsgaard S. (2014). A Kenward-Roger approximation and parametric bootstrap methods for tests in linear mixed models — the R package pbkrtest. J. Stat. Soft. 59, 1–32. 10.18637/jss.v059.i09

[B29] Hintze J. L. Nelson R. D. (1998). Violin plots: a box plot-density trace synergism. Amer. Stat. 52, 181–184.

[B30] Holmqvist K. Nyström M. Andersson R. Dewhurst R. Jarodzka H. van de Weijer J. (2011). Eye Tracking: A Comprehensive Guide to Methods and Measures. Oxford: Oxford University Press.

[B31] Hwang H. Kaiser E. (2014). The role of the verb in grammatical function assignment in English and Korean. J. Exp. Psychol. 40, 1363–1376. 10.1037/a0036797 24884649

[B32] Iwasaki N. (2011). Incremental sentence production: observations from elicited speech errors in Japanese, in Processing and Producing Head-Fina Lstructures, Vol. 38, eds Yamashita H. Hirose Y. Packard J. L. (Dordrecht: Springer), 131–151. 10.1007/978-90-481-9213-7_7

[B33] Jaeger T. F. (2008). Categorical data analysis: away from ANOVAs (transformation or not) and towards logit mixed models. J. Mem. Lang. 59, 434–446. 10.1016/j.jml.2007.11.007 19884961 PMC2613284

[B34] Kalénine S. Mirman D. Middleton E. L. Buxbaum L. J. (2012). Temporal dynamics of activation of thematic and functional knowledge during conceptual processing of manipulable artifacts. J. Exp. Psychol. 38, 1274–1295. 10.1037/a0027626 22449134 PMC3537173

[B35] Kempen G. Hoenkamp E. (1987). An incremental procedural grammar for sentence formulation. Cogn. Sci. 11, 201–258. 10.1207/s15516709cog1102_5

[B36] Kempen G. Huijbers P. (1983). The lexicalization process in sentence production and naming: indirect election of words. Cognition 14, 185–209. 10.1016/0010-0277(83)90029-X

[B37] Kenward M. G. Roger J. H. (1997). Small sample inference for fixed effects from restricted maximum likelihood. Biometrics 53, 983–997. 10.2307/2533558 9333350

[B38] Konopka A. E. Kuchinsky S. E. (2015). How message similarity shapes the timecourse of sentence formulation. J. Mem. Lang. 84, 1–23. 10.1016/j.jml.2015.04.003

[B39] Konopka A. E. Meyer A. S. (2014). Priming sentence planning. Cogn. Psychol. 73, 1–40. 10.1016/j.cogpsych.2014.04.001 24838190

[B40] Kratzer A. (1996). Severing the external argument from its verb, in Phrase Structure and the Lexicon, Vol. 33, eds Rooryck J. Zaring L. (Dordrecht: Springer), 109–137.

[B41] Kuchinsky S. E. (2009). From Seeing to Saying: Perceiving, Planning, Producing. Ph.D. thesis, University of Illinois, Urbana-Champaign, IL.

[B42] Kuchinsky S. E. Bock K. Irwin D. E. (2011). Reversing the hands of time: changing the mapping from seeing to saying. J. Exp. Psychol. 37, 748–756. 10.1037/a0022637 21534707 PMC3087166

[B43] Levelt W. J. M. (2013). A History of Psycholinguistics: The Pre-Chomskyan Era. Oxford: Oxford University Press.

[B44] Lindsley J. R. (1975). Producing simple utterances: How far ahead do we plan? Cogn. Psychol. 7, 1–19. 10.1016/0010-0285(75)90002-X

[B45] Mirman D. (2014). Growth Curve Analysis and Visualization Using R. Boca Raton, FL: Chapman & Hall/CRC.

[B46] Mirman D. Dixon J. A. Magnuson J. S. (2008). Statistical and computational models of the visual world paradigm: growth curves and individual differences. J. Mem. Lang. 59, 475–494. 10.1016/j.jml.2007.11.006 19060958 PMC2593828

[B47] Momma S. Slevc L. R. Phillips C. (2016). The timing of verb selection in Japanese sentence production. J. Exp. Psychol. 42, 813–824. 10.1037/xlm0000195 26569434

[B48] Myachykov A. Tomlin R. S. Posner M. I. (2005). Attention and empirical studies of grammar. Linguist. Rev. 22, 347–364. 10.1515/tlir.2005.22.2-4.347

[B49] Norcliffe E. Konopka A. E. (2015). Vision and language in cross-linguistic research on sentence production, in Attention and Vision in Language Processing, eds Mishra R. K. Srinivasan N. Huettig F. (New Delhi: Springer), 77–96.

[B50] Norcliffe E. Konopka A. E. Brown P. Levinson S. C. (2015). Word order affects the time course of sentence formulation in Tzeltal. Lang. Cogn. Neurosci. 30, 1187–1208. 10.1080/23273798.2015.1006238

[B51] Olsen A. (2012). Tobii I-VT Fixation Filter: Algorithm Description. Technical Report, Tobii Technology, Danderyd.

[B52] R Core Team (2015). R: A Language and Environment for Statistical Computing. Vienna: R Foundation for Statistical Computing.

[B53] Sassenhagen J. Alday P. M. (2016). A common misapplication of statistical inference: nuisance control with null-hypothesis significance tests. Brain Lang. 162, 42–45. 10.1016/j.bandl.2016.08.001 27543688

[B54] Sauppe S. (2017). Symmetrical and asymmetrical voice systems and processing load: Pupillometric evidence from sentence production in Tagalog and German. Language, 93, 288–313. 10.1353/lan.2017.0015

[B55] Schriefers H. Teruel E. Meinshausen R. (1998). Producing simple sentences: results from picture–word interference experiments. J. Mem. Lang. 39, 609–632. 10.1006/jmla.1998.2578

[B56] Shannon C. E. (1948). A mathematical theory of communication. Bell Syst. Tech. J. 27, 379–423. 10.1002/j.1538-7305.1948.tb01338.x

[B57] Sison C. P. Glaz J. (1995). Simultaneous confidence intervals and sample size determination for multinomial proportions. J. Amer. Stat. Assoc. 90, 366–369. 10.1080/01621459.1995.10476521

[B58] Swets B. Jacovina M. E. Gerrig R. J. (2014). Individual differences in the scope of speech planning: evidence from eye-movements. Lang. Cogn. 6, 12–44. 10.1017/langcog.2013.5

[B59] van de Velde M. Meyer A. S. Konopka A. E. (2014). Message formulation and structural assembly: Describing “easy” and “hard” events with preferred and dispreferred syntactic structures. J. Mem. Lang. 71, 124–144. 10.1016/j.jml.2013.11.001

[B60] van Nice K. Y. Dietrich R. (2003). Task sensitivity of animacy effects: evidence from German picture descriptions. Linguistics 41, 825–849. 10.1515/ling.2003.027

[B61] Villacorta P. J. (2012). MultinomialCI: Simultaneous Confidence Intervals for Multinomial Proportions According to the Method by Sison and Glaz. R Package Version 1.0.

[B62] Wickham H. (2009). ggplot2: Elegant Graphics for Data Analysis. Dordrecht; Heidelberg; London; New York: Springer.

[B63] Wundt W. (1900). Völkerpsychologie. Eine Untersuchung der Entwicklungsgesetze von Sprache, Mythus und Sitte. Erster Band: Die Sprache. Leipzig: Wilhelm Engelmann.

