# Supplementary material for: Word Order and Voice Influence the Timing of Verb Planning in German Sentence Production
Source: Front Psychol. 2017 Sep 26;8:1648. doi: 10.3389/fpsyg.2017.01648 (PMC5623055; doi:10.3389/fpsyg.2017.01648)
Supplement: Supplementary file 1 [file Table1.DOCX]

Supplementary Material

Word order and voice influence the timing of verb planning in German sentence production

Sebastian Sauppe*

*** Correspondence:** Sebastian Sauppe, sauppe.s@gmail.com

# Analyses of onset latencies of verbs/auxiliaries following the subject and of second NPs

Table S1: Results from linear mixed effects regression model predicting log-transformed onset latencies of verbs (V-medial actives) or auxiliaries (V-final actives, passives) following the subject

|  | β | \|t\| | F statistic | p |
| --- | --- | --- | --- | --- |
| Intercept | 7.87 | 242.51 |  |  |
| Actives vs. Passives | 0.03 | 1.01 | *F*(2, 24) = 1.55 | 0.23 |
| V-final Actives vs. V-medial Actives | -0.04 | 1.46 |  |  |

Table S2: Results from linear mixed effects regression model predicting log-transformed onset latencies of second NP (patient in actives and agent in passives)

|  | β | \|t\| | F statistic | p |
| --- | --- | --- | --- | --- |
| Intercept | 7.75 | 238.86 |  |  |
| Actives vs. Passives | -0.02 | 0.92 | *F*(2, 23) = 0.49 | 0.62 |
| V-final Actives vs. V-medial Actives | 0.02 | 0.60 |  |  |

# Target stimulus pictures

* – primarily elicited V-medial active sentences; ¶ – primarily elicited V-final active sentences; † – primarily elicited passive sentences; stimulus pictures in parentheses were excluded from analysis

1. Ambulance car colliding with a woman^†^
2. Baker kneading bread dough*
3. Bird pulling a worm out of the ground*
4. Boxer beating a man*
5. Boy breaking branch from a tree*
6. Boy catching a frog*
7. Boy eating corn*
8. Boy kicking a football*
9. Boy kicking against a rock*
10. Boy stirring in a soup*
11. Bull attacking a girl*
12. Cat catching a mouse*
13. Cat scratching a girl’s knee*
14. Construction worker losing his hat*
15. Cowboy catching a bull with a lasso*
16. Crocodile biting into a man’s leg*
17. Dog chasing a mailman^†^
18. Dog chasing a squirrel*
19. Dog sniffing on a mandarin*
20. Frog eating a fly*
21. Gardener planting a tree*
22. Girl hanging out laundry*
23. Girl opening a door*
24. (Girl playing with a jumping rope)
25. Girl pushing a boy*
26. (Girl running towards an open door)
27. Girl tripping a construction worker*
28. Lion eating a dead zebra^¶^
29. Man angling a fish^¶^
30. Man breaking a piece of wood with a hammer^¶^
31. Man chopping a log of wood^¶^
32. Man cutting wood*
33. Man kicking against a chair*
34. Man leaving a hut*
35. Man pushing a car*
36. Man throwing a baby up in the air*
37. Monkey holding a crab in its hand*
38. Mosquito stinging a football player^†^
39. Mouse nibbling on a chocolate bar*
40. Nurse washing a baby*
41. Old man opening a window*
42. Old man reading a book*
43. Old woman climbing up the stairs*
44. Owl carrying a bag*
45. Paper boy selling newspapers*
46. Police officer arresting a man*
47. Police officer stopping a sports car*
48. Police officer stopping a walker-by*
49. Pupil raising his hand*
50. Rabbit eating a carrot*
51. Sailor drinking from a bottle*
52. Sheep eating leaves from a bush*
53. Soldier shooting a man*
54. Train colliding with a bus*
55. Veterinarian examining a horse’s hoof*
56. Woman lifting a rug*
57. Woman looking inside a basket*
58. Woman walking across a bridge*


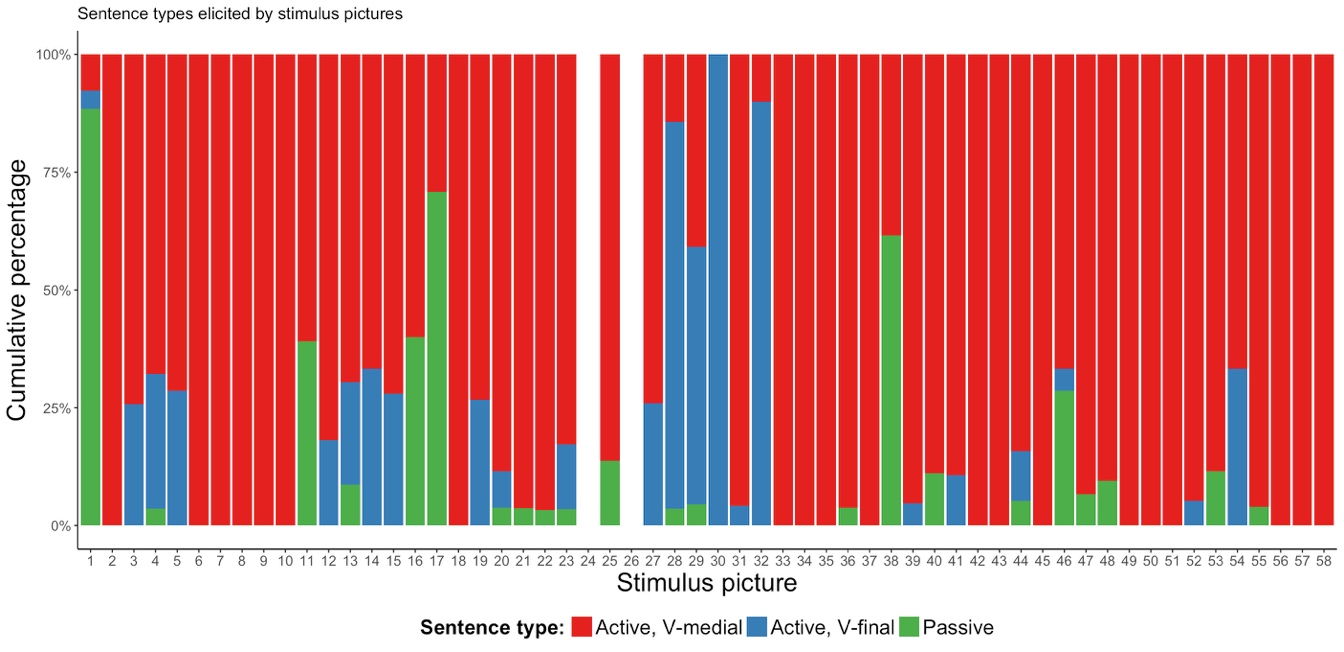


Figure S1: Proportions of sentence types elicited by stimulus pictures.

# Proportions of fixations


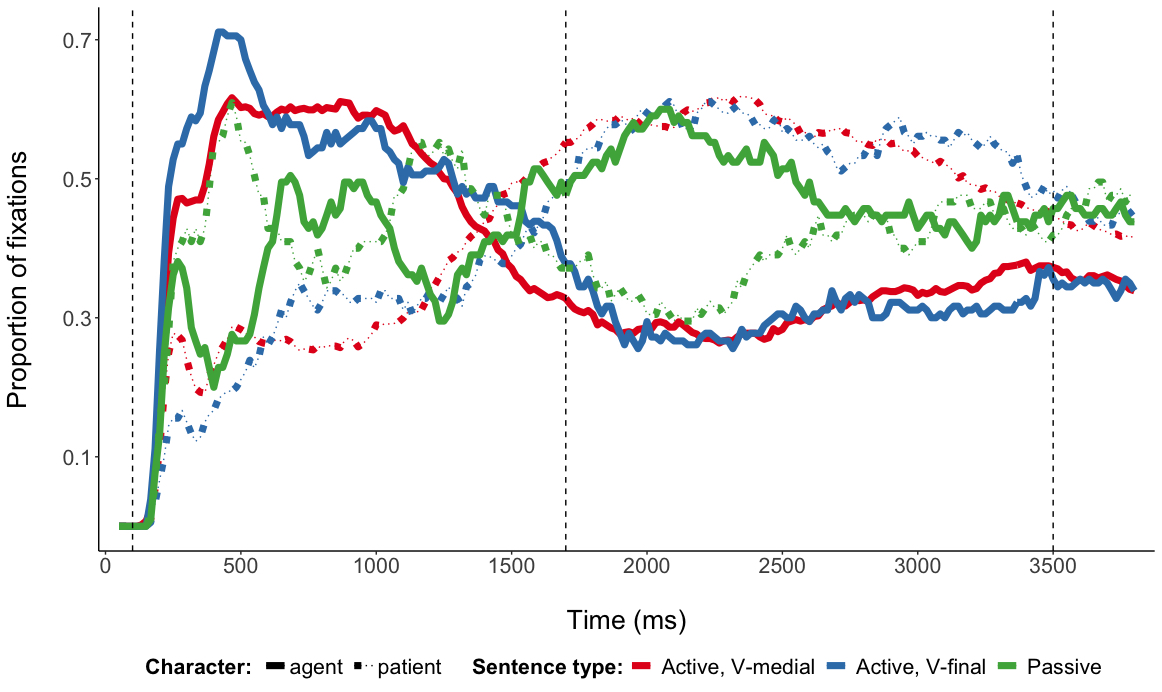


Figure S2: Proportion of fixations to agents and patients during the production of three German sentence types. Vertical lines indicate analysis time windows.

# Model fits


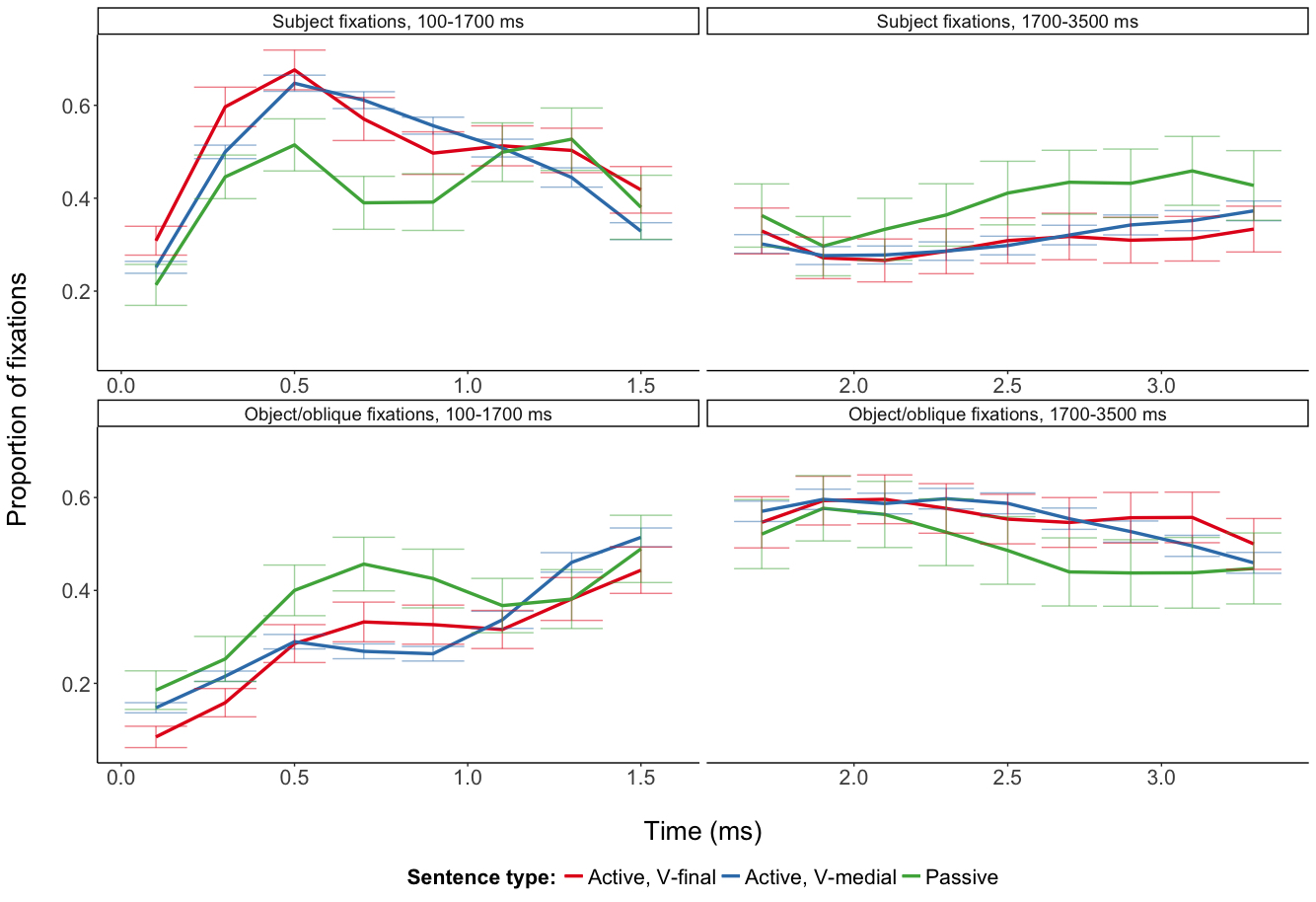


Figure S3: Mean fitted values from logistic mixed effects regression models predicting fixations on subject and object/oblique characters in three German sentence types. Error bars indicate one standard error of the mean fitted values.
